# Tobacco Smoking and Associated Factors Among People Living With HIV in Uganda

**DOI:** 10.1093/ntr/ntaa262

**Published:** 2021-06-08

**Authors:** Noreen Dadirai Mdege, Fredrick Edward Makumbi, Ronald Ssenyonga, Frances Thirlway, Joseph K.B. Matovu, Elena Ratschen, Kamran Siddiqi, Kellen Nyamurungi Namusisi

**Affiliations:** 1 Department of Health Sciences, Faculty of Sciences, University of York, York, UK; 2 Department of Epidemiology and Biostatistics, School of Public Health, College of Health Sciences, Makerere University, Kampala, Uganda; 3 Department of Sociology, Faculty of Social Sciences, University of York, York, UK; 4 Department of Community & Public Health, Faculty of Health Sciences, Busitema University, Mbale, Uganda; 5 Department of Disease Control and Environmental Health, School of Public Health, College of Health Sciences, Makerere University, Kampala, Uganda; 6 Hull York Medical School, University of York, Heslington, York, UK; 7 Department of Health Policy Planning and Management, School of Public Health, College of Health Sciences, Makerere University, Kampala, Uganda

## Abstract

**Introduction:**

The prevalence of smoking among people living with HIV (PLWH) in Uganda is high.

**Aims and Methods:**

We assessed the smoking patterns, behaviors, and associated factors among PLWH in Uganda through a cross-sectional survey. Descriptive statistics were used to describe smoking patterns and behaviors. Logistic regression was used to identify factors associated with current smoking status.

**Results:**

We recruited 777 participants between October and November 2019: 387 (49.8%) current smokers and 390 (50.2%) nonsmokers. 60.9% were males, and the mean age was 40.5 (SD 10.7) years. In multivariate logistic regression, the following increased the odds of being a current smoker: being male (odds ratio [OR] 6.60 [95% confidence interval, CI = 4.34–10.04]), having at least two smokers among five closest friends (OR 3.97 [95% CI = 2.08–7.59]), living in smoking-permitted households (OR 5.83 [95% CI = 3.32–10.23]), alcohol use (OR 3.96 [95% CI = 2.34–6.71]), a higher perceived stress score (OR 2.23 [95% CI = 1.50–3.34]), and higher health-related quality of life (OR 5.25 [95% CI = 1.18–23.35]). Among smokers, the mean Fagerström Test for Nicotine Dependence score was 3.0 (SD 1.9), and 52.5% were making plans to quit. Self-efficacy to resist smoking and knowledge of the impact of smoking on PLWH’s health were low.

**Conclusions:**

Being male, having at least two smokers among five closest friends, living in smoking-permitted households, alcohol use, higher perceived stress scores, and higher health-related quality of life were associated with being a current smoker. Smokers had low to moderate nicotine dependence, high willingness to quit, and low self-efficacy.

**Implications:**

Future behavioral smoking cessation interventions for PLWH should address co-consumption with alcohol and comorbid mental health conditions that are common among PLWH such as stress. In addition, they should take into account the lack of knowledge among this population of the impact of smoking on their health, and low self-efficacy. Given the relatively low levels of nicotine dependency and high levels of willingness to quit in our sample, smoking cessation interventions, if offered, are likely to support this population in achieving long-term smoking abstinence.

## Introduction

With antiretroviral therapy (ART), people living with HIV (PLWH) can have a near normal life expectancy.^1^ However, smoking is a key cause of excess morbidity and premature mortality in this population.^2^ Nearly a quarter of deaths among PLWH on ART are attributable to smoking.^3^ PLWH, on average, lose 12.3 life-years if they smoke: more than twice the number of life-years lost to HIV alone.^4^ In Sub-Saharan Africa, the prevalence of smoking among PLWH is generally higher than that among HIV-negative individuals^5^: it is, on average, one and a half times higher in men living with HIV than HIV-negative men, and almost twofold in women living with HIV than HIV-negative women.^6^ In Uganda, the prevalence of smoking among PLWH is 20% for men and 6% for women,^7^ compared with 10% and 2% for general population men and women, respectively.^8^ Low- and middle-income countries have been recommended to integrate tobacco use cessation into their HIV programs to address comorbidities and improve health outcomes for PLWH.^9^ However, there is a lack of evidence to guide policy and practice on this matter.

Systematic reviews have found that smoking cessation interventions with established effectiveness for the general population are also effective among PLWH but only in the short term (<6 months); there is a lack of evidence on effective interventions to achieve long-term abstinence.^10–12^ One of the cited reasons for this lack of effectiveness is that these interventions do not address the key modifiable smoking determinants, including smoking triggers and relapse predictors, common among PLWH who smoke.^10,11^

Whilst some high-income country studies have characterized smoking behaviors among PLWH and factors contributing to the high smoking prevalence in this population,^13,14^ these are not well documented in Africa. In this paper, we report the smoking patterns and behaviors of PLWH who are in HIV care in Uganda and highlight the factors associated with their smoking status. An understanding of these smoking determinants will help in designing bespoke behavioral interventions that may achieve long-term smoking abstinence in this population.

## Methods

### Study Design

The study was a cross-sectional survey informed by the COM-B model which denotes a “behaviour system” in which capability (C), opportunity (O), and motivation (M) interact to generate behaviour (B).^15^ The individual’s psychological and physical capabilities to engage in the activity concerned, influence their behavior.^15^ For successful long-term quitting, individuals need the relevant knowledge and skills. Automatic brain mechanisms and processes that energize and direct behavior also influence behavior.^15^ For example, an individual’s perceptions of their susceptibility to the harms of smoking^16,17^ can influence their motivation to quit and smoking behavior.^17^ Other physical or social opportunities that make smoking possible or prompt it^15^ may play a bigger part: eg, the opportunity to smoke anywhere, or having many close acquaintances who smoke. The COM-B model was used to determine key domains to include in the questionnaire (Supplementary Table T1).

### Study Sites

We randomly selected eight out of the 15 Uganda Demographic Health Surveys (DHS) regions for 2016.^18^ The smoking prevalence across the eight regions (ie, Ankole, Acholi, Bugisu, Busoga, Kampala, Lango, South Central, and Tooro) ranged from 3.8% to 16.7% for men, and 0.3% to 1.1% for women.^18^ From each of these eight regions, we randomly selected one district (Supplementary Table T2).^18^ Thus, eight out of the 112 Uganda DHS districts for 2016^18^ were included. For each of the eight districts, we used information on ART-accredited health facilities from Uganda’s HIV treatment guidelines,^19^ and information from District Health Officers, to select one to three high patient volume HIV clinics from which study participants were recruited.

### Study Sample

We recruited a convenience sample of PLWH from 16 participating HIV clinics between October 1 and November 30, 2019. To be eligible, participants had to be aged ≥18 years, and able to complete the survey in local languages or in English. We estimated that we would be able to enroll approximately 750 participants (375 current smokers and 375 nonsmokers) over the 2 months recruitment period. This would allow us enough power to be able to make comparisons between the current smokers and nonsmokers, within a 4% margin of error with associated 95% confidence levels.^20^ Sample sizes of this magnitude have been found to increase the accuracy of estimates and represent the desired parameters in the targeted population through simulations, clinical data applications and rule of events per variable approaches.^21–23^

### Procedures

#### Study Approvals

Ethics and other approvals for the study protocol and materials were obtained from the Makerere University School of Public Health Higher Degrees, Research and Ethics Committee (protocol number HDREC704), Uganda National Council for Science and Technology (approval number SS5073), and the University of York’s Health Sciences Research Governance Committee.

#### Recruitment and Enrollment

Clinic staff from the participating clinics informed potential participants about our study and referred those who were interested to research assistants who screened them to determine study eligibility. Those who were eligible were given written study information in English, or in a local language. In the case of literacy problems, the research assistants also provided the information verbally, in the presence of a witness if required. Research assistants obtained written informed consent from all study participants.

#### Measures

We collected sociodemographic data (date of birth, sex, education, and marital status) using questions from Uganda’s DHS.^18^ Self-reported data on number of years since HIV diagnosis, ART status, and number of years on ART were also collected. We collected data on the ownership of 15 household assets (ie, electricity, flush toilet, latrine, fixed telephone, cell phone, television, radio, refrigerator, car or truck, moped/scooter/motorcycle, animal drawn cart, boat with motor, boat without a motor, computer, cassette/cd/dvd player)^18^ from which a wealth index was derived. Asset-based wealth indices are commonly used in low- and middle-income countries, although they might not provide a true representation of consumption expenditure, particularly where only a low proportion of consumption is captured by the included assets.^24^

Current tobacco smoking status was assessed using Global Adult Tobacco Survey (GATS) questions.^25^ A participant was classified as a current smoker if they answered “daily” or “less than daily” to the question “*Do you currently smoke tobacco on a daily basis, less than daily, or not at all?”* We collected data, from all participants, on the following psychosocial and clinical variables that have been identified in the literature as potentially contributing to the high smoking prevalence and difficulties in quitting among PLWH: alcohol consumption using the Alcohol Use Disorders Identification Test (AUDIT-C), a three-item alcohol screen^26,27^; cannabis use using the three cannabis use questions from the ASSIST-Lite^26,28^—cannabis is the most widely used and primary illicit psychoactive substance of concern in Uganda^29^; stress using the 14-item Perceived Stress Scale^13,14,30^; depression using the Patient Health Questionnaire-9 (PHQ-9), a nine-item questionnaire scored from 0 to 27, with a higher score indicating more severe depressive symptoms^13,14,31^; and anxiety using the General Anxiety Disorders-7 (GAD-7), a seven-item instrument scored from 0 to 21, with a higher score indicating more severe anxiety.^13,14,32^ Health-related quality of life (HRQoL) was assessed using the EQ-5D-3L which characterizes health on five dimensions (mobility, self-care, ability to undertake usual activities, pain/discomfort, anxiety/depression).^33,34^ Smoking restrictions at home, number of smokers in the participant’s household, and number of close friends who smoke were assessed using GATS questions^25^ and those suggested by Moran et al.^35^ For smoking restrictions at home, participants were stratified into two groups— those who lived in homes that permitted smoking indoors (ie, smoking-permitted homes), and those that did not (ie, smoke-free homes). Similarly, participants were stratified into two groups according to whether smoking in front of children was permitted or not. Details on how these stratifications were achieved are provided in Supplementary Material for methods. All study participants completed GATS questions that assess the use of smokeless tobacco.^25^

For current smokers, the following information was also collected:

- Types of tobacco product smoked, smoking frequency and quantity, and age on initiation.
- Nicotine dependency from smoked tobacco using the Fagerström Test for Nicotine Dependence (FTND).^36^
- Reasons for smoking using the Attitudes Towards Smoking Scale (ATS-18)^37^ and the Risk Perception Scale.^38^
- Quit intentions, motivation, and behaviors using questions from Fava et al.^39^ based on the stages of change model. These included whether the participant was seriously thinking about quitting or planning to quit smoking; and whether they had a quit attempt during the 12 months prior to survey completion.
- For those who had visited a health care provider in the past 12 months, we assessed whether they were asked about their smoking status, advised to quit, or received smoking cessation support or referral for such support.
- Capacity to stop smoking using the Self-Efficacy Questionnaire (SEQ-12)^40^ and the Multidimensional Scale of Perceived Social Support.^41^
- Whether they mainly smoked alone or with other people.

More detailed descriptions of the AUDIT-C, ASSIST-Lite, EQ-5D-3L, Perceived Stress Scale, PHQ-9, GAD-7, FTND, ATS-18, Risk Perception Scale, SEQ-12, and Multidimensional Scale of Perceived Social Support are provided in Supplementary Material for methods.

Those who were current smokers and had consumed alcohol in the past 3 months were also asked about how often they smoked cigarettes while drinking alcohol, and what happened to their smoking levels when they were drinking.^42^

The questionnaire was designed to take ~60 minutes to complete.

#### Data Collection

Data were collected by 14 trained research assistants. A questionnaire was developed, translated to the appropriate non-English languages and piloted among ~10 participants before being used for the survey. The survey was interviewer-administered face-to-face using Open Data Kit (ODK) (https://getodk.org/) on mobile phones. Missing data and inconsistencies were checked at the end of each survey, and before the participant left the interview whenever possible.

#### Participant Reimbursement

Participants did not receive any financial incentives or reimbursement but were offered a snack whilst completing the study procedures.

#### Data Analysis

For the analysis, the districts were grouped into the following geographical regions: East, North, West, and Central. Principal component analysis was used to generate a wealth index for each participant by identifying a smaller number of uncorrelated variables from the data on ownership of the 15 household assets and using these to create a principal component score.^43^ This score was then used to categorize participants into five wealth-quintiles (lowest, lower, middle, high, and highest).

For descriptive analysis, variables were summarized and presented as percentages for categorical variables and means (SDs) or medians (interquartile range) for continuous variables.

A multilevel mixed-effects logistic regression model was fitted, adjusting for the lowest clustering variable, which was the HIV clinic, to identify factors associated with the dependent variable, current smoking status (ie, current smoker or nonsmoker). Odds ratios (ORs) were used as the measures of association. First, bivariate analysis was conducted to determine the empirical relationship between each independent variable and the dependent variable. All factors with *p* < .2 in the bivariate analysis,^26^ and those that were known, theoretically or empirically, to be associated with smoking status were included in the multivariate analysis. We tested for multicollinearity using the Variance Inflation Factor. The model goodness-of-fit was determined using the Hosmer–Lemeshow test, while the “best” model from competing models was selected using the Akaike’s information criteria.

In the bivariate analysis, the following variables had a *p* value of <.2 and were included in multivariate analysis: sex, number of years on ART, number of smokers among five closest friends, living in a smoke-free/smoking-permitted household, alcohol consumption in the past 3 months, cannabis use in the past 3 months, use of other psychoactive substances in the past 3 months, perceived stress score, and HRQoL (Tables 1–3). Age and level of education were also included in the multivariate analysis because of their strong association with current smoking status in low- and middle-income countries.^44^

“Years since HIV diagnosis” and “living in a household where smoking in front of children is/is not permitted” which had *p* values of <.2 in the bivariate analysis, were not included in the multivariate analysis because of high collinearity with “number of years on ART,” and “living in a smoke-free/smoking-permitted household,” respectively. “Number of smokers in the household” was also not included in the multivariate analysis despite a *p* value of <.2 because of high collinearity with “number of smokers among five closest friends” and poor model fit when this variable was included. Statistical significance was determined at a 5% level. Analyses were conducted in STATA version 14.1.

## Results

### Participant Characteristics

A total of 857 potential participants were screened for study eligibility. Six potential participants did not provide consent, and 74 were ineligible (40 were waiting their confirmatory HIV test results, 32 were <18 years of age, and two could not participate for other reasons). Seven hundred and seventy-seven (90.7%) were eligible for, and consented to participate in, the study (Supplementary Figure F1; Supplementary Table T2).

Three hundred and eighty-seven (49.8%) study participants were current smokers and 390 (50.2%) were nonsmokers (330 never smokers and 60 former smokers). 60.9% of the sample were male, and the mean age was 40.5 (SD 10.7) years. The mean number of years since HIV diagnosis was 7.4 (SD 5.9). Seven hundred and seventy-five (99.7%) were receiving ART and the mean number of years on ART was 6.4 (SD 5.0). Detailed sociodemographic and other participant characteristics are shown in Tables 1–3.

**Table 1:** General Participant Characteristics

Characteristics	Overall (*n* = 777)	Current smokers (*n* = 387)	Nonsmokers (*n* = 390
Age in years: mean (SD)	40.5 (10.7)	40.5 (10.3)	40.5 (11.1)
Sex: *n* (%)^†^			
Female	304 (39.1)	69 (17.8)	235 (60.3)
Male	473 (60.9)	318 (82.2)	155 (39.7)
Years since HIV diagnosis: mean (SD)^†^	7.4 (5.9)	7.0 (5.8)	7.8 (5.9)
Participants on ART: *n* (%)	775 (99.7)	386 (99.7)	389 (99.7)
Years on ART: mean (SD)^†^	6.4 (5.0)	6.1 (5.0)	6.7 (5.0)
Marital status: *n* (%)			
Single/never married	72 (9.3)	38 (9.8)	34 (8.7)
Married/living with partner	433 (55.7)	214 (55.3)	219 (56.2)
Ever married (single due to marital disruption)	272 (35.0)	135 (34.9)	137 (35.1)
Region: *n* (%)			
East	192 (24.7)	95 (24.6)	97 (24.9)
North	203 (26.1)	98 (25.3)	105 (26.9)
West	194 (25.0)	96 (24.8)	98 (25.1)
Central	188 (24.2)	98 (25.3)	90 (23.1)
Education: *n* (%)			
None	76 (9.8)	31 (8.0)	45 (11.5)
Primary	434 (55.9)	219 (56.6)	215 (55.1)
Secondary/tertiary/university/vocational	267 (34.4)	137 (35.4)	130 (33.3)
Wealth index: *n* (%)			
Lowest	245 (31.5)	132 (34.1)	113 (29.0)
Lower	141 (18.2)	73 (18.9)	68 (17.4)
Middle	131 (16.9)	69 (17.8)	62 (15.9)
High	114 (14.7)	51 (13.2)	63 (16.2)
Highest	146 (18.8)	62 (16.0)	84 (21.5)

ART = antiretroviral therapy.

† *p* < .2 in the bivariate analysis.

**Table 2:** Use of Tobacco, Alcohol, Cannabis, and Other Psychoactive Substances

Characteristics	Overall (*n* = 777)	Current smokers (*n* = 387)	Nonsmokers (*n* = 390)
Number of smokers in the household: mean (SD)^†^	0.99 (1.5)	1.6 (1.5)	0.35 (1.1)
Smokers among five closest friends: *n* (%)^†^			
0	379 (48.8)	100 (25.8)	279 (71.5)
1	122 (15.7)	70 (18.1)	52 (13.3)
2+	276 (35.5)	217 (56.1)	59 (15.1)
Smoke-free/smoking-permitted home: *n* (%)^†^			
Smoke-free home	548 (70.5)	204 (52.7)	344 (88.2)
Smoking-permitted home	229 (29.5)	183 (47.3)	46 (11.8)
Smoking in front of children permitted: *n* (%)^†^			
No	331 (42.6)	111 (28.6)	220 (56.4)
Yes	446 (57.4)	276 (71.3)	170 (43.6)
Smokeless tobacco use: *n* (%)			
Not at all	745 (95.9)	369 (95.4)	376 (96.4)
Daily or less	32 (4.2)	18 (4.7)	14 (3.6)
Alcohol consumption: *n* (%)^†^			
No	352 (45.3)	97 (25.1)	255 (65.4)
Yes	425 (54.7)	290 (74.9)	135 (34.6)
AUDIT-C^‡^ (>2 female, >3 male)			
Lower risk	151 (35.5)	99 (34.1)	52 (38.5)
High risk	274 (64.5)	191 (65.9)	83 (61.5)
ASSIST-Lite score (cannabis): *n* (%)^†^			
0	736 (94.7)	351 (90.7)	385 (98.7)
1+	41 (5.3)	36 (9.3)	5 (1.3)
Use of other psychoactive substances: *n* (%)^†^			
No	751 (96.7)	368 (95.1)	383 (98.2)
Yes	26 (3.4)	19 (4.9)	7 (1.8)

AUDIT-C = Alcohol Use Disorders Identification Test.

† *p* <.2 in the bivariate analysis.

‡ The score is based on the 425 who had drunk alcohol in the past 3 months.

**Table 3:** Stress, Anxiety, Depression, and Health-Related Quality of Life (HRQoL)

Characteristics	Overall (*n* = 777)	Current smokers (*n* = 387)	Nonsmokers (*n* = 390)
PHQ-9: *n* (%)			
None_minimal	232 (29.9)	106 (27.4)	126 (32.3)
Mild	206 (26.5)	106 (27.4)	100 (25.6)
Moderate	167 (21.5)	83 (21.5)	84 (21.5)
Moderately severe	103 (13.3)	57 (14.7)	46 (11.8)
Severe	69 (8.9)	35 (9.0)	34 (8.7)
GAD-7: *n* (%)			
None_minimal	279 (35.9)	128 (33.1)	151 (38.7)
Mild	246 (31.7)	126 (32.6)	120 (30.8)
Moderate	136 (17.5)	73 (18.9)	63 (16.2)
Severe	116 (14.9)	60 (15.5)	56 (14.4)
Perceived Stress Score: mean (SD)^†^	2.0 (0.6)	2.06 (0.55)	1.94 (0.58)
HRQoL: mean EQ-5D-3L score (SD)^†^	0.85 (0.17)	0.86 (0.15)	0.84 (0.18)
EQ-5D Visual Analogue Scale: mean (SD)	71.74 (19.0)	71.1 (19.1)	72.4 (18.9)

GAD-7 = General Anxiety Disorders-7; PHQ-9 = Patient Health Questionnaire-9.

† *p* < .2 in the bivariate analysis.

### Smoking Patterns and Behaviors Among Current Smokers

For the 387 current smokers, the mean age of smoking initiation was 20.4 (SD 6.8) years. 79.6% were daily smokers. The most common type of tobacco product smoked was manufactured cigarettes (smoked by 76.7% of participants), followed by hand-rolled cigarettes (12.7% of participants) and pipes full of tobacco (7.8%). The average number of manufactured cigarettes smoked per smoking day was 5.2 (SD 5.6), and this was 3.6 (SD 3.2) for hand-rolled cigarettes, and 3.2 (SD 4.1) for pipes full of tobacco (Supplementary Table T3).

#### Nicotine Dependence

The mean FTND score was 3.0 (SD 1.9). Only 1.3% of the smokers exhibited signs of high nicotine dependence, whilst 22% were moderately dependent, 27.9% were low to moderately dependent, and 48.8% had low dependence.

#### Reasons for Smoking

From the ATS-18, the proportion who agreed/fully agreed that smoking is extremely dangerous to their health, or is ruining their health, was 91.7% and 84.5%, respectively (Supplementary Table T4). 86.3% agreed/fully agreed that smoking leaves an unpleasant smell; and this was 80.4% and 70.6% for the statements that smoking gives them bad breath, and that they spend too much money on cigarettes, respectively. The majority agreed/fully agreed that their cigarette smoke bothers other people a great deal (84.3%), their secondhand smoke was dangerous to those around them (85.5%), smoking was bad for their skin (56.6%), it bothers them to be dependent on cigarettes (62%), and that they would have more energy if they did not smoke (65.6%). The majority agreed/fully agreed that a cigarette calms them down when stressed (72.3%) or when upset (71.6%), and helps them deal with difficult situations (62.8%) or concentrate better (61.3%). The majority agreed/fully agreed that they like the motions of smoking (54.8%), it feels good for them to smoke (64.6%) and they love smoking (55%). 42.6% agreed/fully agreed that they like to hold a cigarette between their fingers.

Using the Risk Perception Scale, only about 43.6% felt that their overall health had been affected by smoking (Supplementary Table T5). The majority thought they were likely or very likely to develop the following in the future if they continued smoking: cancer (71.8%), heart disease (68.5%), and chronic lung disease (81.6%). The proportion who thought their overall health was worse or much worse than that of the average smoker of their age, or the average nonsmoker of their age, was 56.1% and 56.4%, respectively.

#### Social Smoking

One hundred and two (26.4%) smokers reported mainly being with other people when they smoked in the past 30 days, 206 (53.2%) smoked mainly when alone, and 79 (20.4%) as often by themselves as with others.

#### Concurrent Alcohol Consumption

Two hundred and ninety smokers were also alcohol consumers: 202 (69.7%) of these reported that they always or sometimes smoked cigarettes whilst drinking alcoholic beverages, and 160 (55.2%) reported that their smoking levels increased when they were drinking alcohol. 67.5% expressed mild to extreme difficulty in consuming alcohol without smoking a cigarette.

#### Quit Intentions, Motivation, and Behaviors

One hundred and sixty-one (41.6%) smokers reported they were seriously thinking about quitting in the next 4 weeks, and 125 (32.3%) in the next 6 months. Two hundred and three (52.5%) were planning to quit. Two hundred and twelve (54.8%) indicated they had tried to stop smoking in the past 12 months. Of those who had tried to stop smoking in the past 12 months, 109 (51.4%) had tried to quit without any help during their last quit attempt, 52 (24.5%) had tried with assistance from family, 48 (22.6%) had received assistance from their health care provider, and 25 (11.8%) sought assistance from friends. Only 18 (8.5%) had used smoking cessation medicines.

#### Tobacco Use Screening, Advice, and Support From Health Care Professionals

Three hundred and forty-three (88.6%) smokers had visited a health care provider in the past 12 months. Of these, 273 (79.6%) had been asked if they smoked, and 247 (72.0%) had been given advice to stop smoking. Of the ones who had been advised to stop smoking, 129 (52.2%) had been offered smoking cessation information or support—this is 37.6% of those who had visited a health care provider.

#### Capacity to Stop Smoking

From the SEQ-12, less than 30% of smokers reported that they were absolutely sure they would be able to refrain from smoking for each of the following conditions: when feeling nervous, angry, depressed, very anxious or when thinking about a difficult problem (Supplementary Table T6). The proportion of smokers reporting they were absolutely sure they would be able to refrain from smoking when they feel the urge to smoke, having a drink with friends, celebrating, drinking alcohol, were in the company of other smokers, after a meal or having a coffee or tea was between 33% and 41%.

From the Multidimensional Scale of Perceived Social Support, significant others were the most common source of support, followed by family and then friends (Supplementary Table T7). 65.9% strongly/very strongly agreed that there was a special person who was around when they were in need. 65.1% strongly/very strongly agreed that there was a special person with whom they could share their joys and sorrows, whilst this was only 40.6% when asked about friends. 58.7% and 63.3% of smokers strongly/very strongly agreed that there was a special person who was a source of comfort, and cared about their feelings, respectively. 52.7% of smokers strongly/very strongly agreed that their family really tries to help them, whilst this was only 35.1% for friends. 59.7% indicated they could talk about their problems with their family, whilst this was 34.6% for friends. 52.7% strongly/very strongly agreed that they could get the emotional help and support they need from their family, and 49.6% strongly/very strongly agreed that their family helps them make decisions. Only 35.9% strongly/very strongly agreed that they could count on their friends when things go wrong.

### Factors Associated With Current Smoking Status

In the multivariate analysis, males were more likely to be current smokers than females (OR 6.60 [95% confidence interval, CI = 4.34–10.04], *p* < .001), those with at least two smokers amongst their five closest friends were more likely to smoke than those without smokers amongst their five closest friends (OR 3.97 [95% CI = 2.08–7.59], *p* < .001), and those living in smoking-permitted households were more likely to be current smokers than those living in smoke-free homes (OR 5.83 [95% CI = 3.32–10.23], *p* < .001) (Table 4). In addition, those who had drunk alcohol in the past 3 months were more likely to be current smokers than those who had not (OR 3.96 [95% CI = 2.34–6.71], *p* < .001). A higher perceived stress score (OR 2.23 [95% CI = 1.50–3.34], *p* < .001), or HRQoL (OR 5.25 [95% CI = 1.18–23.35], *p* = .030) increased the odds of being a current smoker.

## Discussion

To the best of our knowledge, this study is the first to generate data on the smoking patterns and behaviors, and factors that are associated with current smoking status among PLWH who are in HIV care in Uganda. Being male, having at least two smokers among five closest friends, living in smoking-permitted households and having consumed alcohol in the past 3 months were associated with being a current smoker. A higher perceived stress score or HRQoL score increased the chances of being a current smoker.

In Sub-Saharan Africa, males are generally more likely to be tobacco smokers than females, including among PLWH.^6,45^ However, evidence suggests that the prevalence of smoking among Sub-Saharan African women is rising, with the difference in smoking prevalence between boys and girls narrowing.^46^ Studies have reported that having a social network of smokers, eg, close friends who smoke, facilitates smoking and is a barrier to smoking cessation among PLWH.^47^ Other studies have also shown that living in a smoking-permitted household increases the chances of smoking and decreases the chances of smoking cessation.^48^ Smoking cessation interventions for PLWH therefore need to consider the social and home environments. This might include harnessing the potential of significant others and family members as sources of support for those making a quit attempt.

As for our study, mental health comorbidities such as stress have been reported elsewhere as increasing the chances of smoking among PLWH.^49^ In addition, other studies have also suggested that alcohol consumption increases the chances of smoking among PLWH.^13,14,26^ Unfortunately, there is a potential for a synergistic negative biochemical interaction between alcohol, tobacco, and the HIV virus itself, because of overlapping disease pathways: they all have direct toxic effects, and result in chronic inflammation and the suppression of the body’s immune system.^50,51^ It is therefore important for smoking cessation interventions for PLWH to consider how to deal with the high co-consumption of tobacco with alcohol observed in this population.

Health problems have been shown to trigger smoking cessation.^52–54^ In addition, a near-death experience of full-blown AIDS can trigger PLWH to commit to “new life,” ^55^ and increase their motivation and intention to quit smoking.^56^ These factors could explain why, for our study, those with a lower HRQoL score where less likely to be current smokers than those with a higher score.

Whilst other studies in Sub-Saharan Africa have reported moderate to high levels of nicotine dependence among PLWH who smoke,^57^ for our study, the levels of nicotine dependence were low to moderate among current smokers. Motivation and willingness to quit smoking were also high in our study population. The Uganda Ministry of Health recommends that all PLWH are screened for smoking, and those who smoke encouraged to quit,^19^ and from our study, health care professionals seem to be asking about smoking and offering cessation advice to PLWH, albeit inconsistently and with no cessation support. This could have contributed to the observed high motivation and willingness to quit smoking. HIV treatment initiation in itself can also increase PLWH’s intention to quit smoking, particularly during the first 3 months of treatment.^7,56^ Thus, ART initiation, and the regular contact that PLWH have with health care, provide key opportunities for smoking cessation interventions. However, self-efficacy to resist smoking was low in this population. This is important as the levels of self-efficacy to resist smoking predict levels of craving to smoke, quitting and relapse, including among PLWH.^40,58,59^

Our participants are most representative of adults in HIV care; thus, our results may only be generalizable to similar populations. Additionally, our study did not conduct an analysis for men and women separately as the aim was to inform which intervention components could be important for PLHW overall. Moreover, previous studies in Sub-Saharan Africa did not find major differences between men and women,^26,60^ except for the use of cannabis which was associated with smoking only among men.^26^ Future studies could explore differences that might exist in terms of smoking behavior and factors associated with smoking between different subgroups of PLWH, particularly where differentiated care is feasible.

We recruited participants from eight randomly selected regions of Uganda to enhance the generalizability of our study findings. In addition, unlike other Sub-Saharan African studies that focused on comparing smokers to nonsmokers,^26,60^ we also explored the characteristics of the smokers such as nicotine dependence, reasons for smoking, and capacity to stop smoking. We assessed a wider range of factors including alcohol consumption and its impact on cigarette consumption among smokers, psychosocial factors such as anxiety, stress, and depression. This information is vital in informing the development of the content of smoking cessation interventions that are tailored for PLWH, but is lacking for low- and middle-income countries.^26^

In conclusion, smoking cessation interventions for PLWH should address the lack of knowledge among PLWH of the impact of smoking on their health in particular, co-consumption with alcohol, and low self-efficacy. In addition, they should consider comorbid mental health conditions such as stress and the possibility of utilizing significant others and family members as sources of support. Given the relatively low levels of nicotine dependency and high levels of willingness to quit among PLWH who smoke, appropriately tailored smoking cessation interventions, if offered, are likely to support this population in achieving long-term smoking abstinence.

## Supplementary Material

Supplementary Material A Contributorship Form detailing each author’s specific involvement with this content, as well as any supplementary data, are available online at https://academic.oup.com/ntr.

## Figures and Tables

**Table 4:** Multivariate Analysis Results Showing Factors Associated With Being a Current Smoker

Characteristics	Unadjusted OR (95% CI)	*p*	Adjusted OR (95% CI)	*p*
Age in years	1.00 (0.99, 1.02)	.997	1.00 (0.98, 1.02)	.954
Sex				
Female	1.00		1.00	
Male	6.99 (4.36, 11.21)	<.001	6.60 (4.34, 10.04)	<.001
Education				
None	1.00		1.00	
Primary	1.48 (0.90, 2.42)	.120	0.69 (0.39, 1.22)	.203
Secondary/tertiary/university/vocational	1.53 (0.45, 3.13)	.243	0.58 (0.28, 1.21)	.145
Years on ART	0.98 (0.96, 0.99)	.036	1.02 (0.98, 1.07)	.351
Smokers among five closest friends				
0	1.00		1.00	
1	3.76 (2.72, 5.19)	<.001	1.75 (0.92, 3.35)	.089
2+	10.26 (5.91, 17.83)	<.001	3.97 (2.08, 7.59)	<.001
Smoke-free/smoking-permitted home				
Smoke-free home	1.00		1.00	
Smoking-permitted home	6.71 (3.80, 11.83)	<.001	5.83 (3.32, 10.23)	<.001
Current alcohol consumption				
No	1.00		1.00	
Yes	5.65 (3.67, 8.69)	<.001	3.96 (2.34, 6.71)	<.001
ASSIST-Lite score (cannabis)				
0	1.00		1.00	
1+	7.9 (2.92, 21.35)	<.001	3.84 (0.88, 16.88)	.075
Use of other psychoactive substances				
No	1.00		1.00	
Yes	2.82 (1.31, 6.09)	.008	1.17 (0.47, 2.96)	.735
Perceived Stress Score	1.47 (1.00, 2.15)	.051	2.23 (1.50, 3.34)	<.001
HRQoL: mean EQ-5D-3L score	2.51 (0.83, 7.54)	.102	5.25 (1.18, 23.35)	.030

The following variables were not included in the multivariate model despite having a *p* value <.2 in the bivariate analysis: “Years since HIV diagnosis” because of collinearity with “Years on ART”; “Number of smokers in the household” because of collinearity with “smokers among five closest friends” and poor model fit; “Smoking in front of children” because of collinearity with “smoke-free/smoking-permitted home.” ART = antiretroviral therapy; CI = confidence interval; HRQoL = health-related quality of life; OR = odds ratio.
