# Supplementary material for: Tobacco Smoking and Associated Factors Among People Living With HIV in Uganda
Source: Nicotine Tob Res. 2020 Dec 9;23(7):1208–16. doi: 10.1093/ntr/ntaa262 (PMC7610955; doi:10.1093/ntr/ntaa262)

Manuscript Title: Tobacco smoking and associated factors among people living with HIV in Uganda

Supplementary Figures

F1: Flow of participants in the study

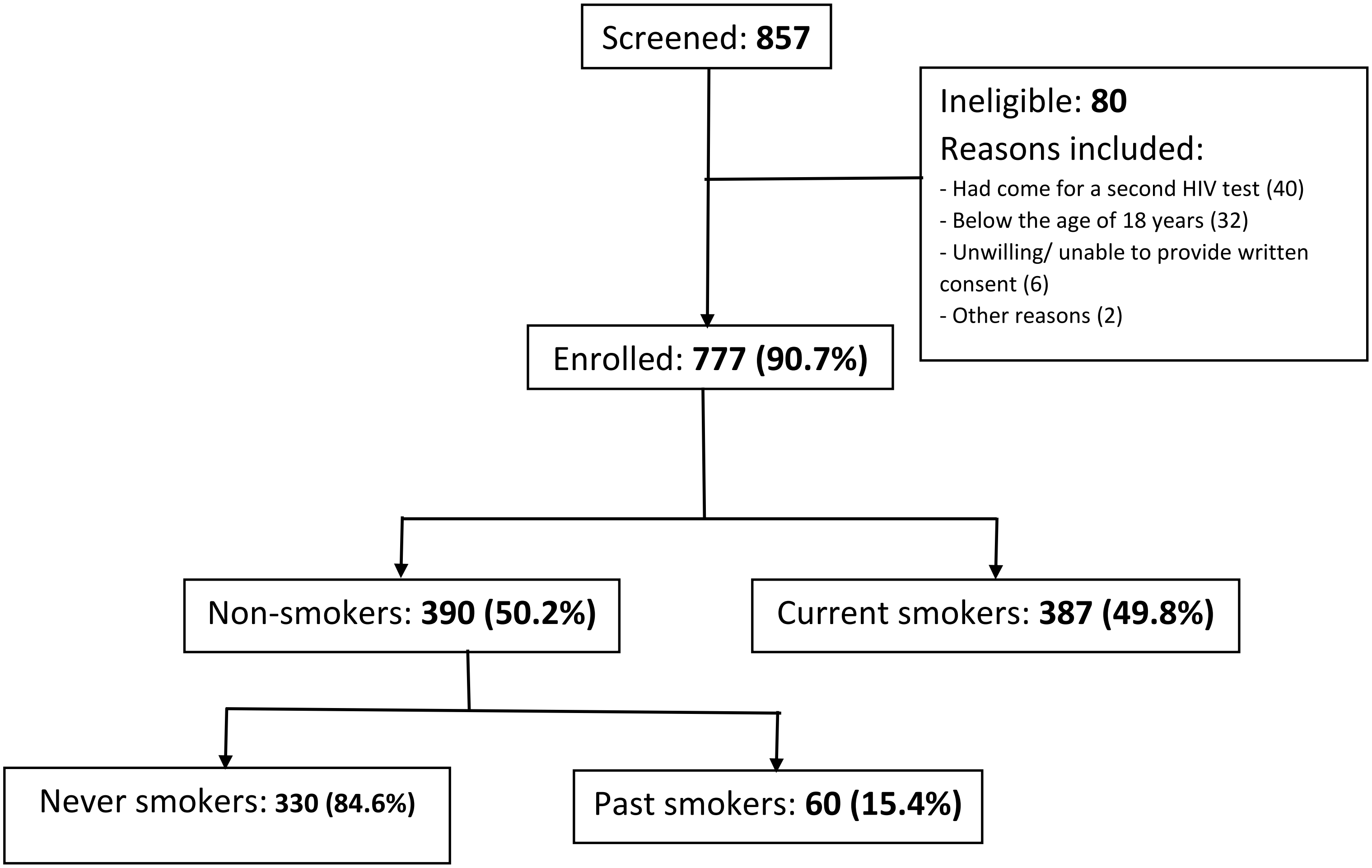

Supplement: ntaa262_suppl_Supplementary_Figures [file ntaa262_suppl_Supplementary_Figures.pdf]
