# Supplementary material for: Tobacco Smoking and Associated Factors Among People Living With HIV in Uganda
Source: Nicotine Tob Res. 2020 Dec 9;23(7):1208–16. doi: 10.1093/ntr/ntaa262 (PMC7610955; doi:10.1093/ntr/ntaa262)
Supplement: ntaa262_suppl_Supplementary_Material_Methods [file ntaa262_suppl_Supplementary_Material_Methods.pdf]

**Manuscript Title: Tobacco smoking and associated factors among people living with HIV in Uganda**

**Supplementary material for methods**

- Stratification to smoke-free home/ smoking-permitted home: SFH status was based on responding ‘only outside’ to two questions: “*Are people who live with you allowed to smoke…?*” and “*Are people who visit your house (including family members), allowed to smoke…*?”. Smoking-permitted homes were those where residents or visitors were allowed to smoke in one part or anywhere inside the house.
- Smoking in front of children: Smoking in front of children was considered as permitted based on responding ‘yes’ or ‘don’t know’ to two questions: “*Are people who live with you allowed to smoke in front of children in the home…?*” and “*Are people who visit your house (including family members), allowed to smoke in front of children in the home…*?”. Any other responses were considered to indicate that smoking in front of children was not permitted.
- The AUDIT-C is a three-item alcohol screen that can help identify persons who are hazardous drinkers or have active alcohol use disorders (including alcohol abuse or dependence).[33] Each item has five answer choices. The AUDIT-C is scored on a scale of 0-12. In men, a score of $\geq$4 or more is considered a positive screen for identifying hazardous drinking or active alcohol use disorders. In women, a score of three or more is considered a positive screen.
- The ASSIST-Lite contains three questions that ask about cannabis use in the past three months.[34] The cannabis score ranges from 0 to three.

- The Perceived Stress Scale is a 14 item scale whose scores are obtained by reversing the scores on the seven positive items, e.g., 0=4, 1=3, 2=2, etc., and then summing across all 14 items.[35] Items 4, 5, 6, 7, 9, 10, and 13 are the positively stated items.
- The PHQ-9 is a nine-item questionnaire which is scored from 0 to 27, and a higher score indicates more severe depressive symptoms.[36] It can also be analysed by grouping individuals in the following categories: none-mild; mild; moderate; moderately severe; and severe.
- The GAD-7 is seven-item instrument scored from 0 to 21, with a higher score indicating more severe anxiety.[37] It can also be analysed by grouping individuals in the following categories: none-mild; mild; moderate; and severe.
- The EQ-5D-3L is a simple standardised generic measure of health-related quality of life developed by the EuroQol Group, where health is characterised on five dimensions (mobility, self-care, ability to undertake usual activities, pain/discomfort, anxiety/ depression).[38,39]
- The Fagerstrom Test for Nicotine Dependence is a standard instrument for assessing the intensity of physical addiction to nicotine which contains six items that evaluate the quantity of cigarette consumption, the compulsion to use, and dependence.[41]
- The Attitudes Towards Smoking Scale (ATS-18) has three subscales that measure perceptions of adverse effects of smoking, the psychoactive benefits and pleasure of smoking.[42]
- The Risk Perception Scale measures the perceived “absolute risk” of smoking (e.g, “What do you think your likelihood is of developing (or if you have, the worsening of) cancer if you continue smoking?”) and “relative risk” perception

(e.g., “How would you compare your overall health to the average smoker your age?”).[43]

- The Self-Efficacy Questionnaire (SEQ-12) consists of two six-item subscales that measure confidence in aptitude to abstain from smoking when facing internal stimuli (e.g. feeling nervous) and external stimuli (e.g. being with a smoker).[45]
- The Multidimentional Scale of Perceived Social Support consists of three subscales assessing support from family, friends, and significant others.[46]
