# Supplementary material for: Tobacco Smoking and Associated Factors Among People Living With HIV in Uganda
Source: Nicotine Tob Res. 2020 Dec 9;23(7):1208–16. doi: 10.1093/ntr/ntaa262 (PMC7610955; doi:10.1093/ntr/ntaa262)
Supplement: ntaa262_suppl_Supplementary_Tables [file ntaa262_suppl_Supplementary_Tables.pdf]

Manuscript Title: Tobacco smoking and associated factors among people living with HIV in Uganda

Supplementary Tables

T1: A summary of variables explored in the cross-sectional survey

**Variables**	**Measures/ Source of questions**
**Socio-demographic and ART status**	
Socio-demographic:<br>• Date of birth, sex, education and marital status, administrative region and district, household assets	Ugandan DHS
Number of years since HIV diagnosis<br>ART status<br>• Current ART status and length of time receiving ART	Study specific questions
**Tobacco use related variables (C- capacity; O- opportunity; M- motivation; B-behaviour)**	
Tobacco smoking **(B)**:<br>• Current smoking status, product type, frequency, and age on	Global Adult Tobacco Survey

initiation	
Nicotine dependence (smoked tobacco)	Fagerstrom Test for Nicotine Dependence
Reasons for smoking **(M)**	Attitudes Towards Smoking Scale (ATS-18)<br>Risk perception scale
Quit intentions, motivation and behaviours **(M)**	Questions from Fava et al (application of the stages of change model); and Global Adult Tobacco Survey
Capacity to stop **(C)**	Self-efficacy questionnaire-12<br>Multidimentional Scale of Perceived Social Support
Physical and social environment **(O)**:<br>• Smoking restrictions at home<br>• Number of smokers in the household<br>• Number of close friends who smoke	Maron et al; Global Adult Tobacco Survey
Smokeless tobacco use:<br>• Current smokeless tobacco use status, product type, frequency, and age on initiation	Global Adult Tobacco Survey

**Other health related variables**	
Alcohol use	AUDIT-C
Use of other substances (Cannabis and other psychoactive substances)	ASSIST-Lite
Health Related Quality of Life	EQ-5D-3L
Stress	Perceived Stress Scale
Depression	Patient Health Questionnaire-9 (PHQ-9)
Anxiety	General Anxiety Disorders-7 (GAD-7)

T2: Regions, districts and clinics from which participants were recruited

Geographic Region	Administrative region	District	Health clinic	Number of participants
East	Bugisu	Mbale	Mbale RRH/TASO	48
			Namataala HC IV	48
	Busoga	Jinja	Jinja RRH/TASO	48
			Walukuba	48
North	Acholi	Gulu	Gulu RRH	51
			Gulu TASO	48
	Lango	Lira	Lira Hospital	49
			PAG Lira	55
West	Ankole	Mbarara	Mbarara RRH	48
			Buzibwera HC IV	49
	Tooro	Kabarole	Virika Hospital	49
			Kabarole RRH	48
Central	Kampala	Kampala	Kisenyi	42
			IDI/Kawala or Komamboga/TASO	48
	South Central	Wakiso	Mildmay	50
			Entebbe Grade B	48

T3: Tobacco products smoked

**Tobacco products**	**Tobacco product smoked**	**Smoked in past 30 days**	**Average days smoked in the past 30 days** mean (SD)	**Average quantity smoked on a smoking day** mean (SD)
All	*387 (100)*			
Manufactured cigarettes	333 (76.7)	333 (76.9)	25.4 (7.6)	5.2 (5.6)
Hand-rolled cigarettes	55 (12.7)	55 (12.7)	19.5 (11.1)	3.6 (3.2)
Kreteks	1 (0.2)	1 (0.2)	30 (0)	1 (0)
Pipes full of tobacco	34 (7.8)	34 (7.9)	25.7 (8.3)	3.2 (4.1)
Cigars, cheroots, or cigarillos	1 (0.2)	1 (0.2)	15 (0)	2 (0)
Number of water pipe sessions	3 (0.7)	2 (0.5)	2 (1.7)	1 (0)
Any others (Kubba, Cannabis, Marijuana, Mijaja sacket)	7 (1.6)	7 (1.6)	20 (10.6)	6.3 (10.6)

T4: Attitude Towards Smoking

	Totally Disagree	Don't really agree	More or less agree	Agree	Fully agree
**ATS-1: Adverse effects of smoking**					
Smoking is extremely dangerous to my health	6 (1.6)	13 (3.4)	13 (3.4)	76 (19.6)	279 (72.1)
Smoking is ruining my health	23 (5.9)	18 (4.6)	19 (4.9)	85 (22.0)	242 (62.5)
My cigarette smoke leaves an unpleasant smell	20 (5.2)	6 (1.6)	27 (7.0)	95 (24.5)	239 (61.8)
Smoking gives me very bad breath	29 (7.5)	8 (2.1)	39 (10.1)	90 (23.3)	221 (57.1)
I spend too much money on cigarettes	28 (7.2)	40 (10.3)	46 (11.9)	80 (20.7)	193 (49.9)
My cigarette smoke bothers other people a great deal	17 (4.4)	10 (2.6)	34 (8.8)	95 (24.6)	231 (59.7)
My second-hand smoke is dangerous to those around me	19 (4.9)	15 (3.9)	22 (5.7)	89 (23.0)	242 (62.5)
Smoking is bad for my skin	81 (20.9)	39 (10.1)	48 (12.4)	75 (19.4)	144 (37.2)
It bothers me to be dependent on cigarettes	49 (12.7)	38 (9.8)	60 (15.5)	86 (22.2)	154 (39.8)
I would have more energy if I did not smoke	54 (14.0)	24 (6.2)	55 (14.2)	96 (24.8)	158 (40.8)
**ATS-2: Psychoactive benefits of smoking**					

A cigarette calms me down when I am stressed	51 (13.2)	16 (4.1)	40 (10.3)	91 (23.5)	189 (48.8)
Smoking calms me down when I am upset	60 (15.5)	17 (4.4)	33 (8.3)	97 (25.1)	180 (46.5)
A cigarette helps me deal with difficult situations	75 (19.4)	17 (4.4)	54 (14.0)	90 (23.3)	151 (39.0)
After a cigarette, I am able to concentrate better	80 (20.7)	19 (4.9)	51 (13.2)	92 (23.8)	145 (37.5)
**ATS-3: Pleasure of smoking**					
I like the motions of smoking	74 (19.1)	41 (10.6)	60 (15.5)	89 (23.0)	123 (31.8)
It feels so good to smoke	47 (12.1)	27 (7.0)	62 (16.0)	98 (25.3)	153 (39.5)
I love smoking	77 (19.9)	35 (9.0)	62 (16.0)	84 (21.7)	129 (33.3)
I like to hold a cigarette between my fingers	135 (34.9)	46 (11.9)	41 (10.6)	60 (15.5)	105 (27.1)

T5: Risk Perception Scale

	**Not at all**	**Not much**	**Neutral**	**To some extent**	**Very much**
To what extent do you feel your overall health has been affected by smoking?	73 (18.9)	63 (16.3)	82 (21.2)	42 (10.8)	127 (32.8)
How much do you feel smoking is responsible for your current illness?	178 (46.0)	67 (17.3)	42 (10.8)	25 (6.5)	75 (19.4)
	**Not at all**	**Not likely**	**Neutral**	**Likely**	**Very likely**
What do you think your likelihood is of developing (or if you have, the worsening of) cancer if you continue smoking?	36 (9.3)	33 (8.5)	40 (10.3)	26 (6.7)	252 (65.1)
What do you think your likelihood is of developing (or if you have, the worsening of) heart disease if you continue smoking?	46 (11.9)	31 (8.0)	45 (11.6)	43 (11.1)	222 (57.4)
What do you think your likelihood is of developing (or if you have, the worsening of) lung disease if you	24 (6.2)	25 (6.5)	22 (5.7)	45 (11.6)	271 (70.0)

continue smoking?					
	**Much worse**	**Worse**	**The same**	**Better**	**Much better**
How would you compare your overall health to the average smoker your age?	95 (24.6)	122 (31.5)	99 (25.6)	29 (7.5)	42 (10.8)
How would you compare your overall health to the average non-smoker of your age?	167 (43.2)	51 (13.2)	48 (12.4)	13 (3.4)	108 (27.9)

T6: Self-efficacy Questionnaire (SEQ-12)

	**Not at all sure**	**Not very sure**	**More or less sure**	**Fairly sure**	**Absolutely sure**
When I feel nervous	111 (28.7)	72 (18.6)	43 (11.1)	51 (13.2)	110 (28.4)
When I feel depressed	108 (27.9)	79 (20.4)	35 (9.0)	52 (29.2)	113 (29.2)
When I am angry	106 (27.4)	75 (19.4)	51 (13.2)	42 (10.8)	113 (29.2)
When I feel very anxious	100 (25.8)	84 (21.7)	48 (12.4)	52 (13.4)	103 (26.6)
When I want to think about a difficult problem	86 (22.2)	88 (22.7)	59 (15.3)	45 (11.6)	109 (28.2)
When I feel the urge to smoke	127 (32.8)	64 (16.5)	41 (10.6)	29 (7.5)	126 (32.6)
When having a drink with friends	121 (31.3)	49 (12.7)	36 (9.3)	28 (7.2)	153 (39.5)
When celebrating something	109 (28.2)	55 (14.2)	43 (11.1)	35 (9.0)	145 (37.5)
When drinking beer, wine, or other spirits	130 (33.6)	46 (11.9)	21 (5.4)	32 (8.3)	158 (40.8)
When I am with smokers	163 (42.1)	43 (11.1)	20 (5.2)	21 (5.4)	140 (36.2)
After a meal	129 (33.3)	63 (16.28)	38 (9.82)	37 (9.56)	120 (31.01)
When having coffee or tea	139 (35.9)	43 (11.1)	34 (8.8)	34 (8.8)	137 (35.4)

T7: Multidimensional Scale of Perceived Social Support

	Very Strongly Disagree	Strongly Disagree	Mildly Disagree	Neutral	Mildly Agree	Strongly Agree	Very Strongly Agree
**Support from a significant other**							
There is a special person who is around when I am in need.	48 (12.40)	10 (2.58)	20 (5.17)	16 (4.13)	38 (9.82)	107 (27.65)	148 (38.24)
There is a special person with whom I can share my joys and sorrows.	32 (8.27)	15 (3.88)	25 (6.46)	9 (2.33)	54 (13.95)	108 (27.91)	144 (37.21)
I have a special person who is a real source of comfort to me.	42 (10.85)	19 (4.91)	27 (6.98)	12 (3.10)	60 (15.50)	94 (24.29)	133 (34.37)
There is a special person in my	34 (8.79)	13 (3.36)	27 (6.98)	12 (3.10)	56	100	145

life who cares about my feelings.					(14.47)	(25.84)	(37.47)
**Support from family**							
My family really tries to help me.	39 (10.08)	15 (3.88)	36 (9.30)	23 (5.94)	70 (18.09)	89 (23.00)	115 (29.72)
I get the emotional help and support I need from my family.	54 (13.95)	14 (3.62)	29 (7.49)	25 (6.46)	61 (15.76)	93 (24.03)	111 (28.68)
I can talk about my problems with my family.	32 (8.27)	11 (2.84)	33 (8.53)	23 (5.94)	57 (14.73)	100 (25.84)	131 (33.85)
My family is willing to help me make decisions.	47 (12.14)	18 (4.65)	46 (11.89)	22 (5.68)	62 (16.02)	92 (23.77)	100 (25.84)
**Support from friends**							
My friends really try to help me.	56 (14.47)	26 (6.72)	64 (16.54)	40 (10.34)	65 (16.80)	86 (22.22)	50 (12.92)

I can count on my friends when things go wrong.	63 (16.28)	31 (8.01)	62 (16.02)	30 (7.75)	62 (16.02)	99 (25.58)	40 (10.34)
I have friends with whom I can share my joys and sorrows.	46 (11.89)	24 (6.20)	63 (16.28)	30 (7.75)	67 (17.31)	85 (21.96)	72 (18.60)
I can talk about my problems with my friends.	53 (13.70)	34 (8.79)	52 (13.44)	42 (10.85)	72 (18.60)	89 (23.00)	45 (11.63)
